# Anemia Increases Oxygen Extraction Fraction in Deep Brain Structures but Not in the Cerebral Cortex

**DOI:** 10.3389/fphys.2022.896006

**Published:** 2022-06-17

**Authors:** Jian Shen, Xin Miao, Chau Vu, Botian Xu, Clio González-Zacarías, Aart J. Nederveen, John C. Wood

**Affiliations:** ^1^ Biomedical Engineering, University of Southern California, Los Angeles, Los Angeles, CA, United States; ^2^ Siemens, Boston, MA, United States; ^3^ Neuroscience Graduate Program, University of Southern California, Los Angeles, Los Angeles, CA, United States; ^4^ Amsterdam UMC, Radiology and Nuclear Medicine, University of Amsterdam, Amsterdam, Netherlands; ^5^ Department of Pediatrics and Radiology, Children’s Hospital Los Angeles, Los Angeles, CA, United States

**Keywords:** sickle cell anemia, oxygen extraction fraction, internal cerebral vein, susceptibility, QSM, CISSCO, trust

## Abstract

Sickle cell disease (SCD) is caused by a single amino acid mutation in hemoglobin, causing chronic anemia and neurovascular complications. However, the effects of chronic anemia on oxygen extraction fraction (OEF), especially in deep brain structures, are less well understood. Conflicting OEF values have been reported in SCD patients, but have largely attributed to different measurement techniques, faulty calibration, and different locations of measurement. Thus, in this study, we investigated the reliability and agreement of two susceptibility-based methods, quantitative susceptibility mapping (QSM) and complex image summation around a spherical or a cylindrical object (CISSCO), for OEF measurements in internal cerebral vein (ICV), reflecting oxygen saturation in deep brain structures. Both methods revealed that SCD patients and non-sickle anemia patients (ACTL) have increased OEF in ICV (42.6% ± 5.6% and 30.5% ± 3.6% in SCD by CISSCO and QSM respectively, 37.0% ± 4.1% and 28.5% ± 2.3% in ACTL) compared with controls (33.0% ± 2.3% and 26.8% ± 1.8%). OEF in ICV varied reciprocally with hematocrit (*r* ^2^ = 0.92, 0.53) and oxygen content (*r* ^2^ = 0.86, 0.53) respectively. However, an opposite relationship was observed for OEF measurements in sagittal sinus (SS) with the widely used T_2_-based oximetry, T_2_-Relaxation-Under-Spin-Tagging (TRUST), in the same cohorts (31.2% ± 6.6% in SCD, 33.3% ± 5.9% in ACTL and 36.8% ± 5.6% in CTL). Importantly, we demonstrated that hemoglobin F and other fast moving hemoglobins decreased OEF by TRUST and explained group differences in sagittal sinus OEF between anemic and control subjects. These data demonstrate that anemia causes deep brain hypoxia in anemia subjects with concomitant preservation of cortical oxygenation, as well as the key interaction of the hemoglobin dissociation curve and cortical oxygen extraction.

## Introduction

Sickle Cell Disease (SCD) is a genetic disorder characterized by a single base pair mutation in the beta subunit of hemoglobin that causes the abnormal hemoglobin S (HbS) to polymerize after deoxygenation leading to chronic hemolytic anemia and neurovascular complications (Ohene-Frempong et al., 1998). SCD patients have an abnormally high and early risk for stroke (Hassell, 2010). The incidence of primary overt stroke has been significantly reduced by Transcranial Ultrasound (TCD) screening and chronic transfusion therapy (Adams, 2005). However, silent cerebral infarction (SCI) is even more common and there is lack of established relationship between SCI presence and TCD measurements (Wang et al., 2000). Imaging of brain oxygenation could be a powerful tool to assess the risk of stroke and aid in its prevention. The oxygen extraction fraction (OEF) has been recognized as an accurate and specific marker for tissue viability and a predictor of misery perfusion in carotid artery disease (Tanaka et al., 2008; Muroi et al., 2011). However, compared with other markers such as cerebral blood flow (CBF), studies on the oxygenation estimation in SCD, especially in deep brain structures, are scarce and results have been conflicting (Jordan et al., 2016; Bush et al., 2017; Fields et al., 2018; Guilliams et al., 2018; Juttukonda et al., 2019). The gold standard for oxygen metabolism is Positron Emission Tomography (PET) imaging (Mintun et al., 1984; Diringer et al., 2007). However, PET is limited by its high cost, invasiveness, long scan time, poor availability, and high exposure to radiation. Therefore, noninvasive estimates of global and regional brain oxygenation are strongly needed.

Currently, T_2_-Relaxation-Under-Spin-Tagging (TRUST) is a widely used MRI technique to quantitatively estimate global brain blood oxygenation via the measurement of pure blood T_2_ (Lu and Ge, 2008; Lu et al., 2012). Unfortunately, TRUST can only provide global saturation for the whole brain without offering oxygenation information in deep brain structures. Furthermore, there exists uncertainty in the proper calibration between T_2_ and oxygen saturation in SCD patients because red blood cell (RBC) morphology and permeability are deranged in these patients (Bush et al., 2021). Unlike T_2_ oximetry, susceptibility-based oximetry (SBO) methods are based on the paramagnetic susceptibility of venous blood. These methods usually measure magnetic susceptibility shift of a vein and there is a linear relationship between magnetic susceptibility shift of blood and concentration of deoxyhemoglobin. Quantitative Susceptibility Mapping (QSM) is a widely used technique to derive a pixel-wise susceptibility map from its induced magnetic field based on the 3D dipole convolution model (Wang and Liu, 2015). Through multiple image processing steps, QSM allows quantification of susceptibility for tissue with arbitrary geometry and orientation, which can be used to estimate oxygen saturation in deep brain structures. An alternative susceptibility-based method called CISSCO (Complex Image Summation around a Spherical or a Cylindrical Object) was introduced to quantify the susceptibility of any narrow cylindrical object at any orientation using a typical multi-echo gradient echo sequence (Cheng et al., 2009; Hsieh et al., 2015a, 2015b). The CISSCO method is based on the complex MR signal whereas QSM calculation is based on the phase signal, and they can be both generated from a typical multi-echo gradient echo scan. Despite the increasing applications of QSM and CISSCO, neither has been used in patients with chronic anemia and *in vivo* validation of these two techniques remains lacking.

The primary purpose of this study was to compare oxygen utilization in deep cerebral structures compared to oxygen saturation from the cerebral cortex. To accomplish this, we performed compared QSM and CISSCO measurements of oxygen saturation in the internal cerebral vein (ICV) with oxygen values derived from TRUST in the sagittal sinus in healthy subjects (CTL), sickle cell anemia patients (SCD) and anemia patients with normal hemoglobin (ACTL). The secondary objective was to cross-validate QSM and CISSCO measurements in the ICV.

## Materials and Methods

### Study Design

This study was approved by our Institutional Review Board (CCI#11-00083) at Children’s Hospital Los Angeles, and all subjects provided written informed consent prior to participation. Data from 28 SCD patients, 18 ACTL patients and 27 healthy control subjects were acquired. Complete blood count, metabolic panel, and hemoglobin electrophoresis were measured at the same day of their MRI scan. Four of the SCD and seven of the ACTL patients were receiving chronic transfusion therapy; these patients were studied on the morning of their transfusion visit prior to transfusion. Genotypes for the SCD patients were SS 25, Sβ+ 1, and SC 3. ACTL patients consisted of thalassemia major 6, hemoglobin H constant spring 3, hemoglobin H disease 2, hereditary spherocytosis 3, Eβ thalassemia major 1, aplastic anemia 1, and autoimmune hemolytic anemia 1. Control subjects were age and ethnicity matched to the SCD population. Eight of these subjects had sickle trait, but prior work from our laboratory has demonstrated indistinguishable cerebral blood flow and brain oxygenation patterns between hemoglobin AA and AS subjects (Vu et al., 2021). The exclusion criteria for all subjects included: 1) pregnancy; 2) hypertension; 3) diabetes; 4) stroke or other known neurologic insult; 5) seizures; 6) known developmental delay or learning disability; and 7) hospitalization within the month prior to the study visit.

Images were acquired on a clinical 3 T Philips Achieva system (Philips Healthcare, Best, Netherlands) with a 32-channel RF coil and a digital receiver chain. The 3D gradient echo sequence had parameters: TR = 30 ms; *α* = 25°; 2 echoes: TE1 = 4.94 ms, ΔTE = 5.2 ms; FOV = 210 * 190 * 120 mm^3^; spatial resolution: 0.6 * 0.6 * 1.3 mm^3^; SENSE acceleration rate = 2 in the phase-encoding direction; BW = 289 Hz/pix and total acquisition time = 6 min 50 s. Flow-compensation was added in the readout direction only, which was the anterior-posterior (AP) direction.

T_2_-Relaxation-under-Spin-Tagging (TRUST) images were acquired from the sagittal sinus as previously described (Lu and Ge, 2008; Miao et al., 2019). Sequence parameters were as follows: TR = 3,000 ms; four effective echoes (eTE) at 0, 40, 80, 160 ms; CPMG τ = 10 m; voxel size = 3.44 * 3.44 mm^2^; FOV = 220 * 220 mm^2^; matrix size = 64 * 64; inversion time (TI) = 1,022 ms and total scan time = 1 min 12 s.

### QSM Processing

For each subject, phase images were fitted to generate a B_0_ field map. Brain extraction and phase unwrapping was performed using FSL (v6.0) (Jenkinson et al., 2012). Background field was removed using projection onto dipole fields (PDF) (Liu et al., 2011). Unreliable phase voxels were identified using the condition of spatiotemporal smoothness of the GRE phase data and removed from the brain mask for subsequent processing (Schweser et al., 2011). L1-regularized field-to-susceptibility inversion was performed to derive the susceptibility map and a weighting parameter λ = $4\times 10^{-4}$ was applied (Bilgic et al., 2012). Venous oxygen saturation (S_v_O_2_) was computed based on:

$$\chi =\left(1-S_{v}O_{2}\right)\chi_{d-o}Hct+\chi_{o-w}Hct,$$ (1)

where χ is the susceptibility measurement of the internal cerebral vein, $\chi_{d-o}$ is the susceptibility shift per unit hematocrit between fully oxygenated and fully deoxygenated erythrocytes, and $\chi_{o-w}$ is the susceptibility shift between oxygenated blood cells and water. Values of 0.27 ppm and -0.03 ppm were used for $\chi_{d-o}$ and $\chi_{o-w}$ (Spees et al., 2001; Langham et al., 2009).

The ROI mask of the internal cerebral vein was manually selected based on the susceptibility map that was threshold at 0.1 ppm to avoid partial-volume effect. The angle between ROI and AP axis was calculated manually from the 3D dataset based on the coordinates of the two end points of the cylinder. Only the segment that had an angle below 30° was included. The purpose was to exclude regions that were susceptible to flow artifact.

### CISSCO Processing

A more detailed description of CISSCO method for susceptibility quantification of a cylindrical object has been presented in (Hsieh et al., 2015a). Here we summarized with the major points and equations. CISSCO integrates the complex MR signals in three annuli around the cylinder of interest. The complex sums were cast into equations containing three unknown parameters, the susceptibility and radius of the vessel, and the proton spin density. The overall MR complex signal S within a coaxial cylinder with radius R was:

$$S=\;\pi l\rho_{0}\vartheta \int_{\vartheta /R^{2}}^{g^{\prime }}\frac{\text{d}x}{x^{2}}J_{0}\left(x\right)+\pi la^{2}\rho_{0,c}e^{i\varphi_{in}},$$ (2)

where a is the vessel radius, the phase value inside the cylinder $\phi_{in}=-\gamma \Delta \chi /6\left(3cos^{2}\theta -1\right)B_{0}T_{E}$ , Δχ  is the susceptibility difference between tissues inside and outside, l  is the slice thickness of the image, $\rho_{0}$ and $\rho_{0,c}$ are the effective spin densities of the tissue outside and inside the object, ϑ is the effective magnetic moment, $g^{\prime }$ = (0.5 $\gamma B_{0}\Delta \chi T_{E})*\mathrm{si}n^{2}\theta$ is the extremum phase value on the surface of the cylinder, θ is the orientation of the cylinder, and J_0_ is the zeroth order Bessel function.

Equation 2 can be applied to all three annuli, allowing us to solve for the three unknown variables; complex signal differences between any two annuli eliminate the second term, eliminating two variables. First, the magnitude and phase images in coronal view were cropped to 64*64 and a 16*16 Gaussian high pass filter was applied to remove background phase. Next, θ was estimated based on the coordinates of the two endpoints of the innermost annuli. The calculated θ was 82.3 ± 5.6°, revealing that the internal cerebral vein is nearly perpendicular to the direction of B_0_. Finally, after applying the equation to three coaxial cylinders, the effective magnetic moment, the effective spin densities, and the susceptibility difference can be solved sequentially.

The resulting Δχ was converted to oxygen saturation using Eq. 1, the same as for QSM.

### TRUST Processing

Control–label difference images for each echo time were averaged and fit to a simple mono-exponential function. In control and ACTL patients, the decay time constant was corrected for T_1_ using an estimated calculated from hematocrit, assuming deoxygenated blood (Lu et al., 2004). In non-transfused SCD patients, venous T_1_ was estimated to be 1818 ms (Václavuu et al., 2016), and for transfused SCD patients T_1_ was estimated using a simple mixture assumption based upon the fraction of circulating hemoglobin S. In control subjects, the resulting T_2_-apparent was converted to oxygen saturation using a calibration derived from human blood (Bush et al., 2018; Li et al., 2020). In SCD patients, a consensus calibration model (Bush et al., 2021) was used to convert T_2_-apparent to oxygen saturation. Separate equations were used for transfused and non-transfused subjects, taking care to correct T_2_-apparent for imperfections in the 180° pulse (Bush et al., 2021).

### Physiological Background

To gain physiological insight into predictors of oxygen saturation in the ICV compared with the sagittal sinus, oxygen extraction fraction (OEF) was calculated separately for the two venous locations (OEF_ICV_, OEF_SS_) as follows:

$$OEF=\frac{\left(S_{a}O_{2}-SvO_{2}\right)}{S_{a}O_{2}},$$ (3)

where $S_{a}O_{2}$ is the arterial saturation measured by pulse oximetry. We compared OEF_ICV_ and OEF_SS_ to O_2_ content using linear regression, with variable transformation when appropriate. O_2_ content was derived as follows, neglecting the impact of dyshemoglobins (Wasserman and Whipp, 1975):

$$O_{2}\;content=1.34*Hb*S_{a}O_{2}.$$ (4)

To provide some physiological background, the equation between O_2_ content, cerebral blood flow (CBF) and cerebral metabolic rate of oxygen (CMRO_2_) is also shown here:

$$CMRO_{2}=OEF*CBF*O_{2}\;content.$$ (5)

Alternatively, Eq. 5 can be recast as follows:

$$OEF=\frac{CMRO_{2}}{\left(CBF*O_{2}\;content\right)}.$$ (6)

Thus, OEF is expected to vary reciprocally with the product of CBF and O_2_ content, which is also referred to as cerebral oxygen delivery.

### Statistical Analysis

Statistical analysis was performed in JMP (SAS, Cary, NC). Demographic and laboratory variables were compared using Analysis of Variance (ANOVA) with Dunnett’s post hoc correction. OEF values derived by QSM and CISSCO (OEF-QSM, OEF-CISSCO) were compared across study groups using ANOVA with Dunnett’s post-hoc correction. Inter-modality comparison was performed using Bland-Altman analysis, with bias assessed using a two-sided, one-sample *t*-Test. Shapiro-Wilks tests of normality were applied to each variable, with transformation, outlier exclusion, or nonparametric testing used when appropriate.

We examined predictors of OEF_ICV_ and OEF_SS_ using linear regression, with variable transformation when appropriate. Predictors included hemoglobin, hematocrit, oxygen saturation, oxygen content, hemoglobin S%, left shifted hemoglobin %, LDH, reticulocyte count, cell free hemoglobin, WBC, MCV, MCH, MCHC, WBC, platelets, and mean platelet volume. All variables with *p* values greater than 0.05 were retained for stepwise regression. Models were built iteratively (two variable models, followed by three variable models, etc), retaining variables yielding the highest combined *r* ^2^. No nonlinear variable interactions were considered.

Given the collinearity between the three strongest predictors (hemoglobin, hematocrit, and oxygen content), we also explored models where one of these three variables was “locked” in the model to inform us about potentially important covariates.

## Results

### Demographics

Among the 73 volunteers participated in the experiment, data from seven subjects were discarded due to motion or low SNR. There were 25 SCD patients, 17 ACTL patients and 24 healthy controls in the final data processing, and the demographics were shown in Table 1. Controls were slightly older than either patient group, but the groups were well balanced for sex. Anemia, corresponding erythropoietic and hemolytic markers, hemoglobin F and total left shifted levels were increased in ACTL and SCD, but more severe in SCD. Oxygen saturation was not different across groups, but diastolic blood pressure was 10% lower in both anemic groups. Forty percent of the ACTL patients were transfused, compared with 16% of SCD, and none of the control subjects.

**TABLE 1:** Subject demographics. Group averages and standard deviations are given.

	Healthy controls	ACTL patients	SCD patients
N	24	17	25
Age	27.3 ± 8.2	21.6 ± 5.6	24.1 ± 6.8
Sex	11F, 13M	10F, 7M	11F, 14M
Hematocrit (%)	40.8 ± 4.1	35.2 ± 5.9*	26.8 ± 4.3*δ
Hemoglobin (g/dl)	13.6 ± 1.4	11.5 ± 2.8*	9.5 ± 1.7*δ
HbS (%)	12.4 ± 17.5	2.5 ± 10.2	67.8 ± 25.2*δ
HbF (%)	0	4.5 ± 6.0*	12.1 ± 10.5*δ
Systolic blood pressure (mmHg)	114.7 ± 9.0	115.2 ± 11.5	114.0 ± 10.0
Diastolic blood pressure (mmHg)	69.0 ± 7.5	62.1 ± 8.1*	61 ± 6.3*
Oxygen Saturation (%)	99.1 ± 1.2	99.2 ± 1.0	98.6 ± 1.4
O_2_ content (ml O_2_/ml blood)	18.5 ± 1.9	15.6 ± 3.8*	12.9 ± 2.4*δ
Lactose dehydrogenase (LDH)	548.2 ± 86.4	635.2 ± 250.0	863.0 ± 432.2*δ
MCV (cubic um/red cell)	85.5 ± 7.4	79.5 ± 9.7	96.1 ± 16.6*δ
MCH (pg/cell)	28.8 ± 2.6	25.8 ± 5.3*	34.0 ± 6.7*δ
MCHC (g Hgb/dl)	33.4 ± 1.3	31.7 ± 4.1	35.2 ± 1.6*δ
Reticulocytes (%)	1.5 ± 0.7	3.6 ± 3.4*	8.4 ± 4.9*δ
Transfused	0/24	7/17*	4/25

*Denotes *p* < 0.05 with respect to control, δ denotes *p* < 0.05 with respect to ACTL.

Table 2 summarizes the Hb, HbS%, HbF%, and OEF values in non-transfused SCD patients with and without hydroxyurea. Patients taking hydroxyurea had higher F%, higher OEF CISSCO, lower S%, and lower sagittal sinus OEF measurements than patients not on hydroxyurea. None of the controls or ACTL patients were taking hydroxyurea.

**TABLE 2:** Differences between hemoglobin F, hemoglobin S and OEF by three methods with or without hydroxyurea (HU) for non-transfused SCD patients.

	With HU	Without HU	*p*-value
Hb	9.5 ± 1.4	12.1 ± 2.7	**< 0.001**
Hb F (%)	18.4 ± 8.8	1.4 ± 3.3	**< 0.001**
Hb S (%)	78.2 ± 8.3	18.2 ± 27.9	**< 0.001**
OEF-TRUST (%)	28.2 ± 6.1	35.3 ± 5.7	**< 0.001**
OEF-CISSCO (%)	42.9 ± 5.5	36.2 ± 5.2	**< 0.001**
OEF-QSM (%)	30.1 ± 4.4	28.0 ± 2.7	0.1034

Bold text depicts a *p*-value < 0.05.

**FIGURE 1:**
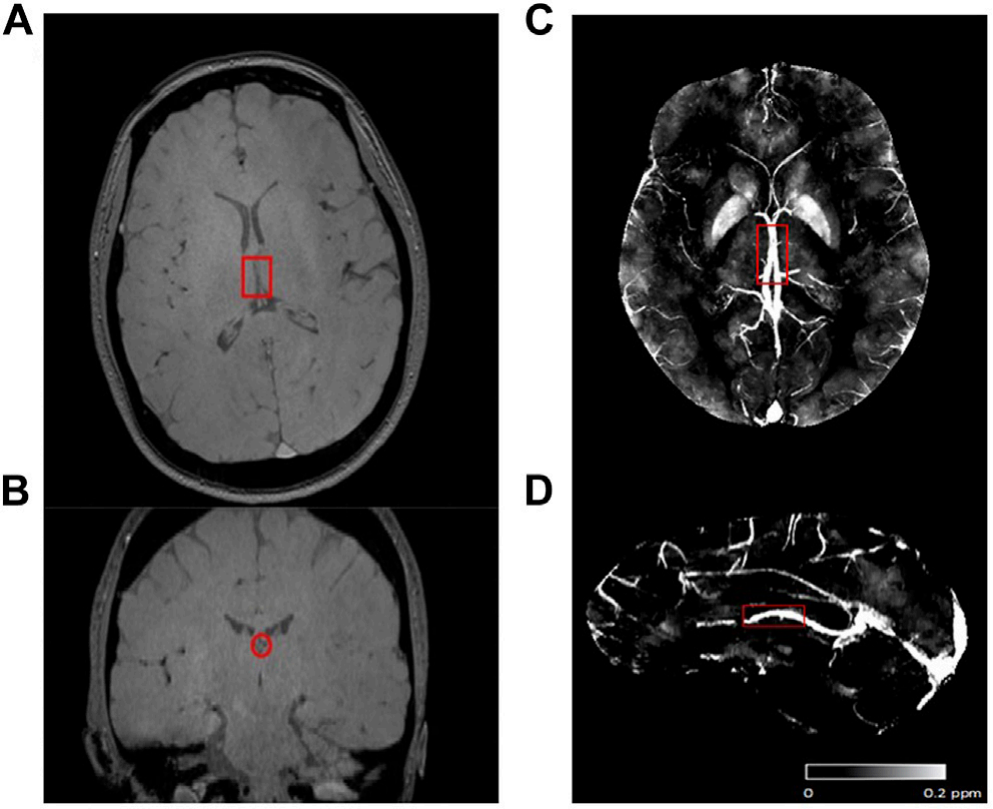
Representative image and region of the internal cerebral vein (ICV, highlight by red rectangle). **(A)** Magnitude in axial view. **(B)** Magnitude in coronal view. **(C)** Axial view, and **(D)** Sagittal view of ROI in the max intensity projection of a representative susceptibility map.

Figure 1A–C show representative magnitude and phase images in both axial and coronal views. The processed susceptibility map by QSM is shown in Figure 1D. ICV generally lies parallel to the axial plane and typically has a quantifiable length around 11 mm by QSM. By CISSCO, the calculated vessel radius, a, was 1.1 ± 0.5 mm and was independent of disease state and hemoglobin level.

### Comparison of OEF Measurements in ICV (by QSM and CISSCO) and SS (by TRUST) for Different Groups

**FIGURE 2:**
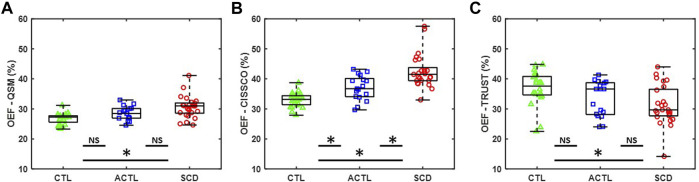
Group differences for OEF measurements by CISSCO, QSM and TRUST. **(A)** Boxplot for OEF-QSM in internal cerebral vein for SCD, ACTL and CTL. **(B)** Boxplot for OEF-CISSCO in internal cerebral vein for SCD, ACTL and CTL. **(C)** Boxplot for OEF-TRUST in sagittal sinus for SCD, ACTL and CTL. (* denoted statistically significant *p* < 0.05; NS denoted no significant difference).

Figure 2A,B summarize the OEF measurements in the internal cerebral vein using QSM (OEF-QSM) and CISSCO (OEF-CISSCO), respectively. Mean OEF-QSM measurements were 30.1% in SCD, 28.3% in ACTL, and 26.6% in CTL (*p* < 0.01). On Dunnett’s post hoc correction, SCD was different from CTL (*p* < 0.001), but ACTL was not (*p* = 0.1069). Mean OEF-CISSCO measurements were 42.5% in SCD, 37.0% in ACTL, and 33.0% in CTL (*p* < 0.01), with both SCD (*p* < 0.001) and ACTL (*p* = 0.007) significantly different from control subjects. Figure 2C shows the OEF measurements in the sagittal sinus vein using TRUST (OEF-TRUST). Mean OEF-TRUST measurements were 31.2% in SCD, 33.4% in ACTL and 36.8% in CTL (*p* < 0.01). After Dunnett’s analysis, we found that SCD was different from CTL (*p* = 0.0034) and ACTL was not (*p* = 0.1291).

**FIGURE 3:**
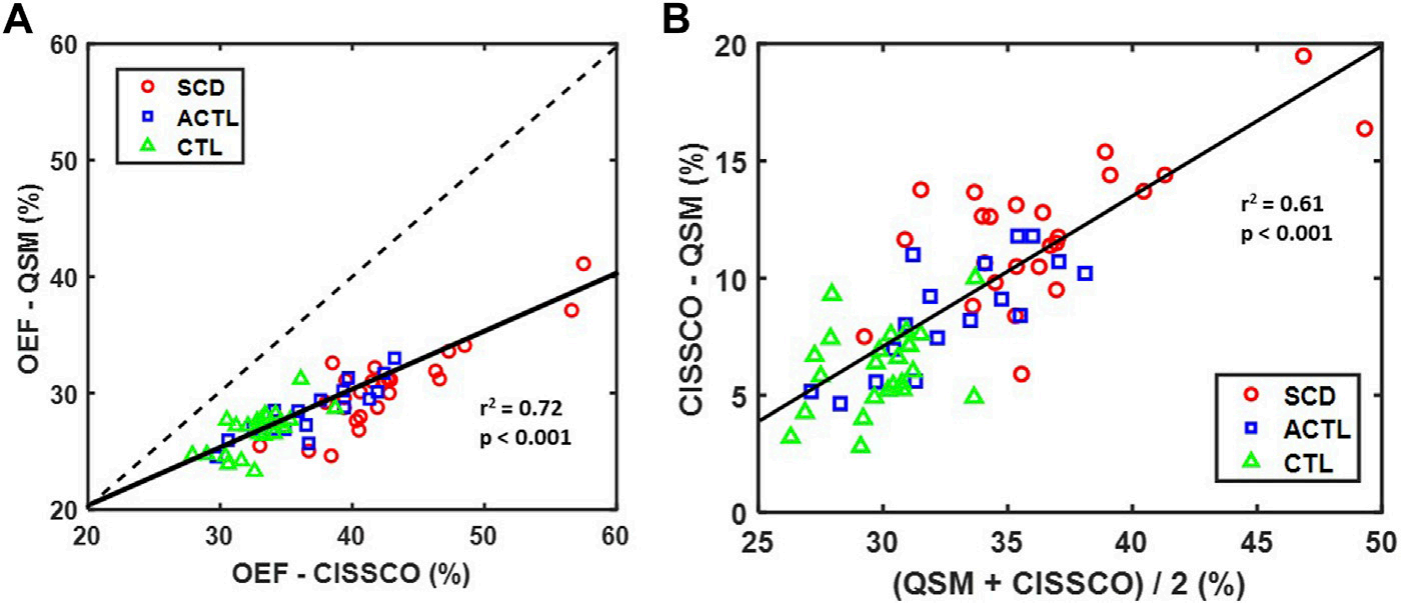
**(A)** Scatter plot of OEF-CISSCO and OEF-QSM with linear correlation line (solid) and identity line (dashed) (*r* ^2^ = 0.72, *p* < 0.001). **(B)** Bland-Altman plot for OEF-CISSCO and OEF-QSM. The linear correlation line (solid) is shown (*r* ^2^ = 0.61, *p* < 0.001).

Figure 3 characterizes the bias between the two OEF_ICV_ measurements using linear correlation (Figure 3A) and Bland-Altman analysis (Figure 3B). A strong linear relationship was observed between OEF-QSM and OEF-CISSCO (*r* ^2^ = 0.72, *p* < 0.001). The mean OEF-CISSCO is 9.3% higher than OEF-QSM (*p* < 0.001). In addition, the bias was proportional to the mean value (*r* ^2^ = 0.61, *p* < 0.001) and was larger in anemic subjects.

### Relationships Between OEF Measurements and O_2_ Content in ICV

**FIGURE 4:**
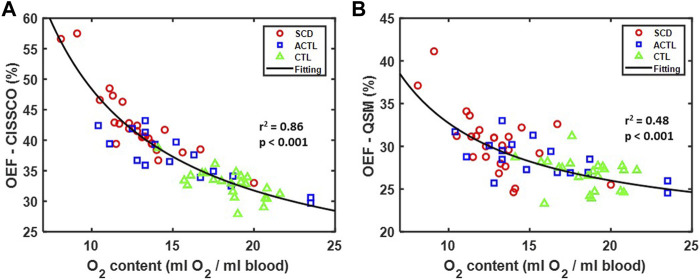
Relationship between OEF and O_2_ content in SCD, ACTL and CTL. **(A)** Scatterplot between OEF-CISSCO and O_2_ content. The fitting reciprocal line is shown in black with *r* ^2^ = 0.86, *p* < 0.001. **(B)** Scatterplot between OEF-QSM and O_2_ content. The fitting reciprocal line is shown in black with *r* ^2^ = 0.48, *p* < 0.001.

The relationship between OEF measurements in the internal cerebral vein and O_2_ content is shown in Figures 4A,B. Both methods demonstrate a reciprocal relationship with O_2_ content, but the variance is significantly less for OEF-CISSCO compared with OEF-QSM (*p* < 0.01 by F test). Importantly, when this relationship is removed, all group differences in OEF disappear for both techniques.

### Relationships Between OEF, Left Shifted Hemoglobin and O_2_ Content in SS.

**FIGURE 5:**
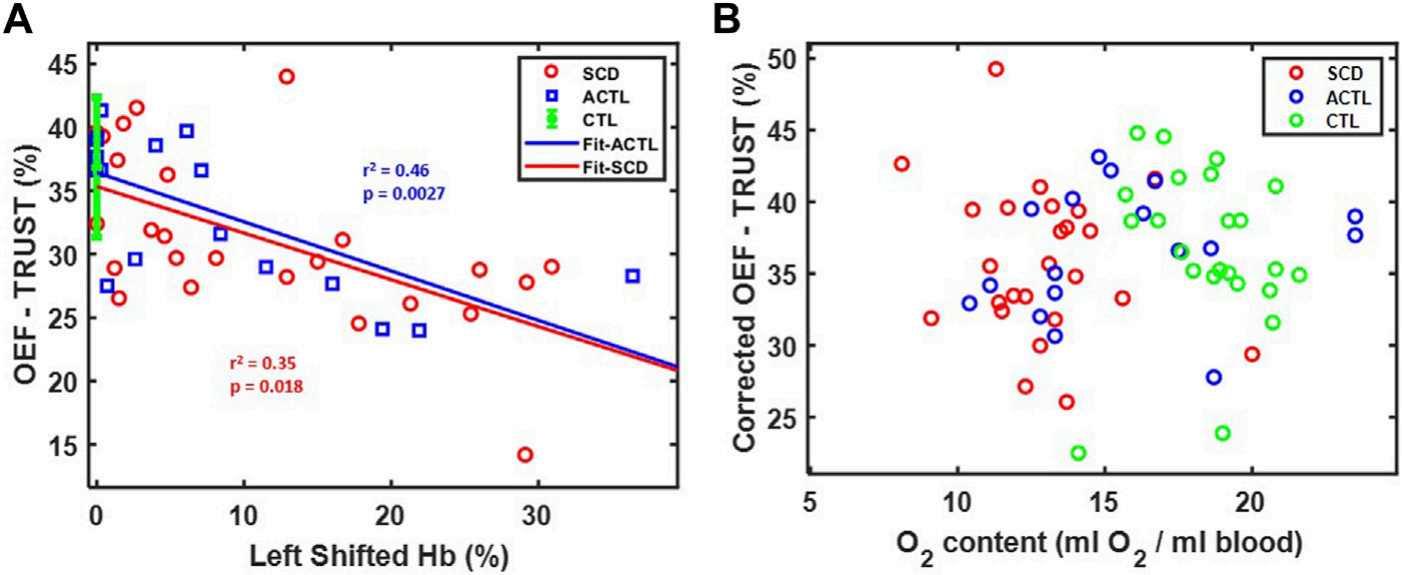
**(A)** Relationship between OEF-TRUST with left shifted hemoglobin. Linear correlations are shown in blue line (*r* ^2^ = 0.46, *p* = 0.0027) for ACTL and red line (*r* ^2^ = 0.35, *p* = 0.0018) for SCD. The control group is shown as 36.8 ± 5.5 (mean ± std) in green. **(B)** Relationship between corrected OEF-TRUST with O_2_ content.

Figure 5A demonstrates OEF-TRUST in the sagittal sinus as a function of left shifted hemoglobin concentration. The left shifted hemoglobin included hemoglobin F in SCD patients and fast moving hemoglobin in ACTL patients with alpha-thalassemia. It revealed that OEF-TRUST in the sagittal sinus declined with increasing left shifted hemoglobin for both SCD (*r* ^2^ = 0.35, *p* = 0.018) and ACTL (*r* ^2^ = 0.46, *p* < 0.0027). The slope was statistically identical in SCD and ACTL, as were the intercepts for all three groups. Thus, after controlling for inter-subject variability in the left shifted hemoglobin, the corrected OEF in the sagittal sinus was independent of group and O_2_ content, as shown in Figure 5B.

### Predictors for OEF in ICV and SS

Table 3 summarizes the primary and secondary predictors for OEF in the ICV and sagittal sinus. For OEF-CISSCO, there were two equivalent models predicting OEF in the ICV with a combined *r* ^2^ of 0.94 and 0.95 respectively. The dominant variable was either oxygen delivery (*r* ^2^ = 0.86), hemoglobin (*r* ^2^ = 0.85) or hematocrit (*r* ^2^ = 0.92), which are intrinsically co-linear (*r* ^2^ = 0.90). To explore what hematocrit reflected, beyond oxygen transport, we locked oxygen content (or hemoglobin alone) into the model, displacing hematocrit but introducing mean corpuscular hemoglobin concentration (MCHC) into the model. If hematocrit entered the model first, hemoglobin, oxygen content, and MCHC were displaced. With either the primary or the alternative model, oxygen saturation by pulse oximetry was positively associated with OEF-ICV.

**TABLE 3:** OEF predictors by stepwise regression.

Residual OEF-CISSCO and O_2_ Content			Residual OEF-TRUST and HbF%		
Parameter	*r* ^2^	*p*-value	Parameter	*r* ^2^	*p*-value
MCHC	0.34	<0.0001	MCH	0.16	0.008
Oxygen Saturation	0.19	0.0003	MCV	0.10	0.03
MCH	0.17	0.001	Height	0.10	0.03
HbS%	0.16	0.009	Weight	0.09	0.05
Weight	0.08	0.03	-		
RBC	0.07	0.04			
MCV	0.07	0.04			
Final Stepwise Model OEF-CISSCO			Final Stepwise Model OEF-TRUST		
1/O_2_ content	0.85	<0.0001	HbF	0.34	0.0001
MCHC	+0.05	<0.0001	MCV	+0.13	0.004
Oxygen Saturation	+0.04	<0.0001	-	-	-
Total *r* ^2^	0.94	-	Total *r* ^2^	0.53	-
Alternative Model OEF-CISSCO			-		
1/Hematocrit	0.92	<0.0001			
Oxygen Saturation	+0.03	<0.0001			
Total *r* ^2^	0.95	-			

After controlling OEF-TRUST for left shifted hemoglobin concentration, there was no residual relationship with patient group, Hb, Hct, RBC or O_2_ content. There were also weak associations with systolic blood pressure (SBP), and the collinear variables MCV and MCH (*r* ^2^ = 0.92 with respect to each other). On stepwise analysis, left shifted hemoglobin, SBP and MCV persisted with a combined *r* ^2^ of 0.47.

## Discussion

In this manuscript, we studied 66 subjects across a broad range of hemoglobin values and identified an increase in OEF in deep brain structures in patients with chronic anemia by two susceptibility-based methods. OEF in the ICV was reciprocally related to hematocrit, which reflected a combination of oxygen content and mean cellular hemoglobin concentration. OEF in the ICV was also directly proportional to peripheral oxygen saturation. In contrast, sagittal sinus OEF was decreased in anemic subjects, proportionally to hemoglobin, hematocrit, and oxygen content. However, after controlling for the impact of left shifted hemoglobin (hemoglobin F, hemoglobin H, hemoglobin Barts), OEF was independent of patient group and oxygen content. OEF by QSM and CISSCO were highly correlated, but QSM yielded systematically lower OEF estimates.

Previous work from our laboratory, and others, has suggested disparate oxygen extraction fraction estimates measured using sagittal sinus oximetry when compared to tissue oximetry performed in deep brain structures (Bush et al., 2018; Croal et al., 2019; Fields et al., 2018). In particular, tissue oximetry suggests profound deep brain hypoxia with worsening anemia (Fields et al., 2018; Guilliams et al., 2018), while sagittal sinus oximetry suggests normal or even decreased oxygen extraction (A. M. Bush et al., 2018; Li et al., 2020). We have previously postulated that a vascular steal phenomenon may exist, where oxygen delivery to brain cortex is preserved at the expense of deep brain structures (Chai et al., 2019). Arterial transit time is decreased in sickle cell disease patients (Afzali-Hashemi et al., 2021), leading to decreased exchange of labeled spins into the cerebrovasculature (A. Bush et al., 2018; Juttukonda et al., 2019). However, this manuscript is the first to document deep brain hypoxia in anemia subjects with concomitant preservation of sagittal sinus oxygen extraction.

The internal cerebral vein is one of the major deep cortical veins, and its oxygen saturation provides insight into the oxygenation of the basal ganglia, corpus callosum and thalamus. OEF estimates using either QSM or CISSCO demonstrated an inverse relationship with O_2_ content (Figure 4), similar to whole brain estimates of OEF by Asymmetric Spin Echo (ASE) (Supplementary Figure S1) (Fields et al., 2018). Although there is a bias between the two datasets, both demonstrate a comparable reciprocal relationship with O_2_ content. The similarity should not be surprising, however, as ASE is dominated by tissue oxygenation in the white matter and deep gray structures; in ASE, much of the signal from cortex is contaminated by susceptibility artifacts from superficial veins and excluded from global OEF measurements. ASE oximetry measurements yield spatially averaged tissue oxygenation that weigh grey matter and white matter equally, despite their markedly disparate contribution to brain metabolism.

According to Eq. 6, OEF may be cast as the ratio of the cerebral metabolic rate of oxygen divided by the product of cerebral blood flow and oxygen content (i.e., O_2_ delivery). On a whole-brain basis, CBF increases reciprocally with O_2_ content in anemic subjects (Borzage et al., 2016; Bush et al., 2016), preserving global oxygen delivery. However, compensatory hyperemia is blunted or eliminated in deep watershed structures (Chai et al., 2019). By Eq. 6, OEF in the ICV (and by ASE) should vary inversely with oxygen content if deep brain blood flow does not augment appropriately.

The other two predictors of deep brain OEF also provide physiological insights. The positive relationship between ICV OEF and S_p_O_2_ arises organically from Eq. 3. The brain operates within a very narrow range of pO_2_, which translates to a much broader range of S_v_O_2_ because of inter-subject variability in the hemoglobin dissociation curve (Wood, 2019). Powerful physiological compensation mechanisms limit declines in S_v_O_2_ under hypoxic conditions. Thus, inter-subject variability in S_a_O_2_, even within the normal range, introduces a positive relationship with OEF by providing more “headroom” for oxygen extraction. The positive association of MCHC and OEF in the ICV is more challenging to explain. MCHC contributes powerfully to RBC deformability and viscosity. We speculate that MCHC could be modulating capillary transit time through its impact on red cell rheology, however this would have to be independently confirmed.

In the sagittal sinus, oxygen saturation is dominated by cortical blood flow and supply-demand matching. Venous oximetry techniques follow Fick-principle (oxygen mass balance) and are inherently flow-weighted rather than spatially weighted. Since grey matter has 3–4 times the metabolic activity of white matter, venous oximetry techniques reflect grey matter oxygen balance. Whole brain CBF rises inversely to oxygen carrying capacity such that oxygen delivery is preserved in anemic subjects (Borzage et al., 2016; Bush et al., 2016). There are also multiple other publications confirming these observations (Prohovnik et al., 2009; Kosinski et al., 2017). Regional flow assessment using ASL demonstrates that cortical oxygen delivery is normal (Chai et al., 2019). Preservation of OEF in the sagittal sinus, with normal or even decreased OEF despite worsening anemia, is the natural consequence of compensatory hyperemia (Bush et al., 2018). The mechanisms behind this “cortical sparing” are unknown but work in mice suggests that chronic hypoxia stimulates cortical capillary proliferation (Harb et al., 2013). Duffin modeled compensatory hyperemia using a “fail-safe” mechanism and proposed potential biochemical mediators (Duffin, 2020). Regardless of the mechanism, observed patterns of brain volume loss (Choi et al., 2019, 2017) and silent infarction (Fields et al., 2018) are consistent with cortical sparing at the expense of the deep watershed areas.

A second striking finding of this study was the powerful effect of hemoglobin F and other high affinity hemoglobin molecules on OEF measured in the sagittal sinus. This undoubtedly represents left-shift of the hemoglobin dissociation curve and resulting higher oxygen affinity of hemoglobin. While this phenomenon is well known, the magnitude of the effect is striking, with OEF decreasing 20 saturation points over the physiologic range of left shifted hemoglobin fraction in anemic subjects. Importantly, all group differences in sagittal sinus OEF were eliminated once differences in high affinity hemoglobin fraction was controlled for. These findings highlight the critical physiologic principle that cerebral OEF is not a regulated variable. The brain varies vascular tone to preserve capillary pO_2_ in a narrow range (Wood, 2019). When hemoglobin is left-shifted, less oxygen is delivered for any brain pO_2_. In SCD patients with high hemoglobin F concentration, resting CBF increases to preserve tissue oxygen unloading in the cortex (Afzali-Hashemi et al., 2021). In contrast, the deep structures have decreased compensatory hyperemia and are forced to operate at lower pO_2_ (increased OEF compounded by tighter oxygen affinity).

So are hydroxyurea or other dissociation curve modifiers placing the deep structures of the brain at risk? The left-shift of hemoglobin cannot be interpreted in isolation. With hydroxyurea, the impaired oxygen unloading is at least partially compensated by a 10%–15% rise in O_2_ content; to date no study has systematically evaluated regional oxygen delivery and metabolism in patients prior to and following hydroxyurea initiation to evaluate that balance between improved oxygen capacity and impaired oxygen unloading. Studies are currently ongoing to explore these endpoints in voxelotor (Vichinsky et al., 2019; Herity et al., 2021), an allosteric modifier of hemoglobin affinity. The present study emphasizes the need to examine both global and regional responses to such therapies.

The powerful impact of HbF and other modulators of the hemoglobin dissociation curve must also be considered when comparing OEF from modern hemoglobinopathy cohorts, with historical data prior to the widespread use of hydroxyurea (Herold et al., 1986; Vu et al., 2021). TRUST studies from modern SCD cohorts (Bush et al., 2017; Afzali-Hashemi et al., 2021; Vu et al., 2021) consistently observed lower OEF values that reported in historical cohorts (Heyman et al., 1952; Herold et al., 1986). Hydroxyurea did not achieve widespread use until the last one to 2 decades. Current recommendations favor introduction at 18 months of age, and dose escalation to maximum tolerated dose, leading to robust hemoglobin F% induction in many patients. In our cohort, OEF was 7% points lower in patients taking hydroxyurea which is consistent with OEF differences exhibited by current and historical cohorts.

It is important to consider whether our two principle findings, i.e. cortical sparing and the powerful effect of left shifted hemoglobin fraction, could be artifacts resulting from the two different oximetry techniques. MRI venous oximetry can be performed using magnetic susceptibility or by R2, R2*, or R2′ relaxometries. If the calibration curves for these methods are unbiased across the patient subgroups, then the techniques can be used interchangeably, and our observed results are valid. The susceptibility calibration is considered the most robust because it is independent of red cell integrity. The linear dependence of susceptibility on hematocrit and oxygen saturation is incontrovertible but estimates of the intrinsic susceptibility of deoxygenated hemoglobin vary from 0.18–0.27 (Weisskoff and Kiihne, 1992; Langham et al., 2009; Jain et al., 2012; Eldeniz et al., 2017; Fields et al., 2018). Errors in this parameter could introduce bias but not alter the direction of change observed. Hemoglobin S has the same intrinsic susceptibility as hemoglobin A (Eldeniz et al., 2017) and there is little biophysical reason to believe that other hemoglobins should be significantly different because the electron shells of the heme moiety are identical (it is just the supporting scaffolding that’s different).

The TRUST calibration in normal subjects (so-called hemoglobin AA calibration) is also fairly incontrovertible. Two independent laboratories have yielded superimposable results over a very broad range of hematocrit values (Bush et al., 2021; Bush et al., 2018; Li et al., 2020). This observation is important because the red blood cells in the ACTL group, and all transfused patients, predominantly contains normal hemoglobin. Results from these patients yield identical findings compared to the SCD patients. Thus, cortical sparing and the effect of left shifted hemoglobin cannot be attributed simply to a faulty oximetry calibration.

The TRUST calibration has challenges in the SCD population that we have characterizing for years (Bush et al., 2021; Bush et al., 2018; Li et al., 2020). The variation in calibration arises from damage to the red cell membrane as well as changes in red cell density and shape (Bush et al., 2021; Bush et al., 2018; Li et al., 2020), not from any intrinsic magnetic difference in sickle hemoglobin (Eldeniz et al., 2017). Since patients with sickle cell trait have normal appearing red blood cells, their blood follows the AA calibration curve, not the SCD curve (Bush et al., 2018). The sickle calibration curve used in this study represents a “consensus” calibration using data pooled from two laboratories to yield a more stable estimate of the hematocrit interaction and to derive a model that accounts for the dilution effect of transfusion (Bush et al., 2021). We believe that the absence of a group effect in Figures 4, 5, despite combining controls, transfused SCD, non-transfused SCD, transfused ACTL, and non-transfused ACTL, offers strong evidence that our TRUST calibrations not introducing systematic bias that could be misinterpreted as physiologic change.

It is also reasonable to wonder about the potential impact of hemoglobin F on the T2 calibration in those patients with excellent response to hydroxyurea (e.g. >15%). The published calibration curve for hemoglobin F cells is quite similar to the HbA calibration (Bush et al., 2017; Liu et al., 2011, p. 2). In SCD patients, hemoglobin F upregulation will cause some degree of hemoglobin F cells to circulate (like hemoglobin A)…this we could potentially compensate for using the mixture model (SS + FF is similar to SS + AA). To determine whether the increased HbF in some of the non-transfused SCD has an important “dilutional” effect, similar to transfusions, we reran all the TRUST data using the mixture model and did not observe any significant qualitative differences to our findings (see Supplementary Figure S2A,B).

In the extremes of hemoglobin F expression (for example after gene therapy or hereditary persistence of fetal hemoglobin), the sickling process is almost completely abrogated and the resulting red cells are morphologically normal. Under this extreme case (which does NOT reflect even the highest F induction in this study), the hemoglobin A calibration would be appropriate. As a result, we also compared the results using the hemoglobin A calibration for all subjects as a “worst case” simulation. When we did this, the two principle findings (cortical sparing, OEF negatively correlated with hemoglobin F) were maintained (Supplementary Figure S2C, D). However, OEF was systematically higher in SCD patients. This is nonsensical because it would imply that cerebral metabolic rate is increased in SCD compared with controls and other anemic subjects. Studies using gold standard techniques like Kety-Schmidt and PET have proven exactly the opposite (see supplemental data in Vu et al., 2021).

Both the CISSCO and QSM methods reveal group differences in venous saturation measurements and similar relationships with hemoglobin. However, there exists significant bias between the two techniques with QSM exhibiting higher saturation values. There might be several reasons for the underestimation of susceptibility for QSM. Firstly, the existence of streaking artifact may affect the measurement of susceptibility indirectly (Li et al., 2015). Secondly, partial-volume effect causes susceptibility to be underestimated, particularly in small vessels like the internal cerebral vein (Zhang et al., 2017). Lastly, QSM estimates of vein saturation are impacted by nonlinear phase accrual in moving spins. This effect could worsen with anemia severity as blood viscosity lessens and blood flow increases. We postulate that the latter two effects are responsible for the systemic bias between QSM and CISSCO oximetry in these patients. CISSCO overcomes these limitations by avoiding unstable dipole inversion and by inferring vessel susceptibility from its effect on surrounding tissue rather than from the blood itself, similar to asymmetric spin echo (ASE).

The CISSCO method is not without its own limitations. CISSCO is most accurate for veins perpendicular to the main magnetic field, making it most effective for the straight sinus and internal cerebral vein. It cannot be used for superficial veins like the sagittal sinus because a complete annulus cannot be drawn across it. CISSCO requires high SNR inside the vessel (over 5:1) for accurate measurements, which limits the choice of echo times and can be impacted by Gibbs ringing (Hsieh et al., 2015a). In practice, we first quantify the magnetic moment from the second echo time as the uncertainty of measurement of magnetic moment at the longer echo time is smaller (Cheng et al., 2009). Then we solve the susceptibility at the first echo time for a higher SNR inside the internal cerebral vein, after scaling the calculated magnetic moment. In addition, improper choice of radius of three annuli might produce up to 15% uncertainty to the calculated susceptibility through error propagation (Hsieh et al., 2015a). However, despite these limitations, the tight relationship of OEF_ICV_ with O_2_ content and the similarity of this relationship with OEF by ASE suggest that it may be a better choice than QSM for venous oximetry in select vessels.

Other alternatives exist to quantify saturation in the deep veins. A variation of TRUST, called TRU-PC (Krishnamurthy et al., 2014), uses complex differencing rather than arterial spin labeling to eliminate partial volume effects and has sufficient signal to quantify T_2_ in deep draining veins. It would be instructive, in the future, to compare OEF estimates by TRU-PC and CISSCO in SCD patients as a mechanism to cross-validate T_2_ and susceptibility-based oximetry in this patient population.

## Conclusion

In summary, we have demonstrated that anemia worsens hypoxia in deep brain structures while cerebral cortex appears to be spared, replicating observations using tissue-based oximetry ASE. Our findings resolve the apparent paradox observed between TRUST and ASE measurements. We also demonstrate that hemoglobin F and other high affinity hemoglobin molecules appear to be powerful modulators of OEF by TRUST and explain group differences in OEF observed between anemic and control subjects. These observations were conserved across diverse study populations, robust with respect to choice of sickle cell calibration, and physiologically plausible.

Although CISSCO was ideal for the present context because is robust to partial volume effects, independent of blood flow velocity, and unaffected by red cell properties, it is only suitable for select cerebral veins, limiting its generalizability. QSM is more generalizable and saturation estimates were qualitatively similar to CISSCO, but troubled by large biases from partial volume and flow effects. Future oximetry in the deep brain may require techniques such as TRU-PC.

## Data Availability

The raw data supporting the conclusion of this article will be made available by the authors, without undue reservation, to qualified investigators following institutional regulatory approval.
