# Supplementary material for: Anemia Increases Oxygen Extraction Fraction in Deep Brain Structures but Not in the Cerebral Cortex
Source: Front Physiol. 2022 Jun 17;13:896006. doi: 10.3389/fphys.2022.896006 (PMC9248375; doi:10.3389/fphys.2022.896006)
Supplement: Supplementary file 1 [file Table1.DOCX]

Anemia Increases Oxygen Extraction Fraction in Deep Brain Structures but Not in the Cerebral Cortex

**Supplemental data**


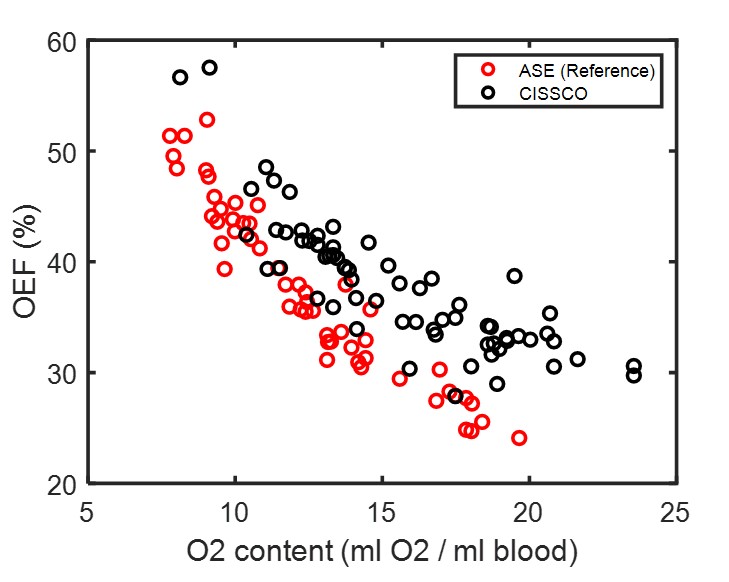


**Figure S1.** Relationship between OEF and O_2_ content by CISSCO (OEF-CISSCO) and reference data (OEF-ASE) (Fields et al., 2018).


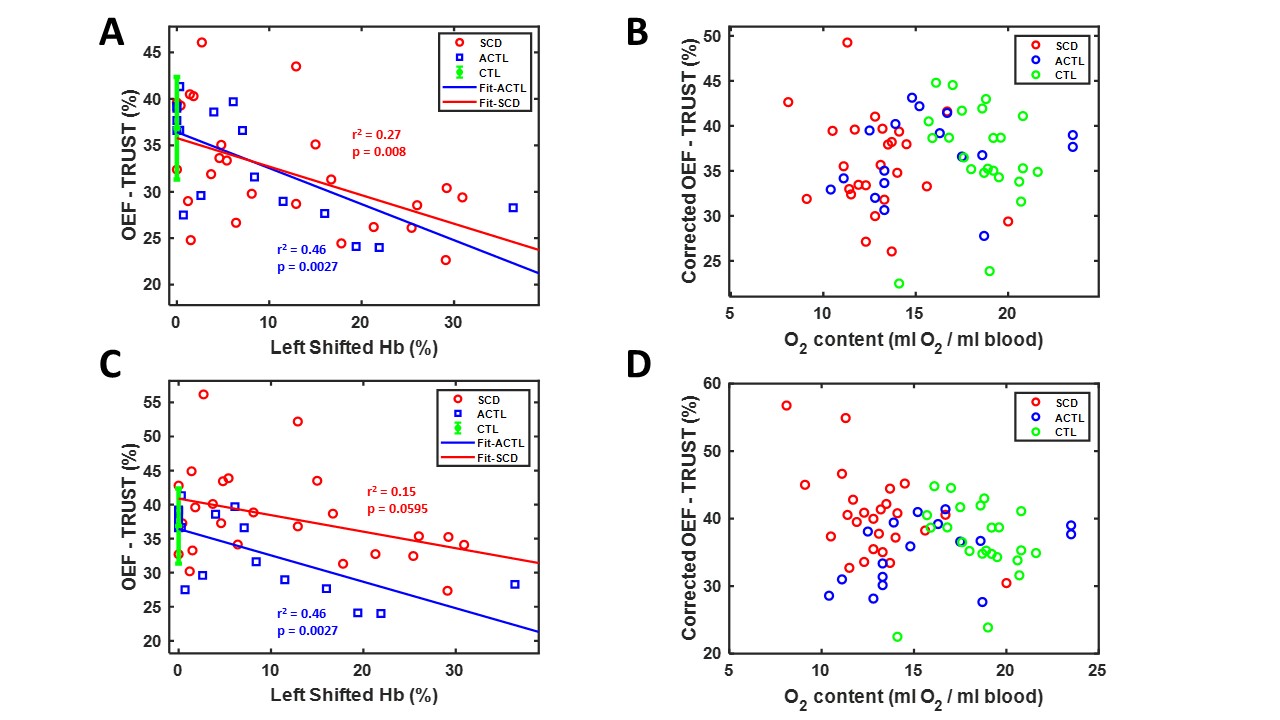


**Figure S2**. **(A)** Relationship between OEF-TRUST with left shifted hemoglobin using the mixture model. Linear correlations are shown in blue line (r^2^ = 0.46, p = 0.0027) for ACTL and red line (r^2^ = 0.27, p = 0.008) for SCD. The control group is shown as 36.8 ± 5.5 (mean ± std) in green**. (B)** Relationship between corrected OEF-TRUST with O_2_ content using the mixture model. Figure S2. **(C)** Relationship between OEF-TRUST with left shifted hemoglobin using the HbA model. Linear correlations are shown in blue line (r^2^ = 0.46, p = 0.0027) for ACTL and red line (r^2^ = 0.15, p = 0.0595) for SCD. The control group is shown as 36.8 ± 5.5 (mean ± std) in green**. (D)** Relationship between corrected OEF-TRUST with O_2_ content using the HbA model.

Reference:

Fields, M.E., Guilliams, K.P., Ragan, D.K., Binkley, M.M., Eldeniz, C., Chen, Y., Hulbert, M.L., McKinstry, R.C., Shimony, J.S., Vo, K.D., others, 2018. Regional oxygen extraction predicts border zone vulnerability to stroke in sickle cell disease. Neurology 90, e1134–e1142.
